# Intraocular Silicone Oil Masquerading as Terson Syndrome

**DOI:** 10.1155/2016/4942109

**Published:** 2016-09-22

**Authors:** Navid Elmi Sadr, Bijan Samavat, Payam Mehrian, Alireza Hedayatfar

**Affiliations:** ^1^Eye Research Center, Rassoul Akram Hospital, Iran University of Medical Sciences, Tehran, Iran; ^2^Telemedicine Research Center, National Research Institute of Tuberculosis and Lung Diseases (NRITLD), Shahid Beheshti University of Medical Sciences, Tehran, Iran; ^3^Noor Ophthalmic Research Center, Noor Eye Hospital, Tehran, Iran

## Abstract

*Introduction*. Terson syndrome is described as intraocular hemorrhage in association with any type of intracranial hemorrhage and is associated with higher mortality rate and vision loss. Intraocular hemorrhage in Terson syndrome may be diagnosed using computed tomography but there are false positive results. Silicone oil which is widely used for internal tamponade of complicated retinal detachments has high attenuation on computed tomography and hyperintensity on T1-weighted magnetic resonance imaging that can mimic intraocular hemorrhage. This report shows that silicone oil is another origin of false positive results in interpreting CT findings for detecting Terson syndrome.* Case Report*. A 71-year-old diabetic woman presented with loss of consciousness. Brain computed tomography revealed right cerebellar hemorrhage and ventricular hemorrhage and hyperdensity in vitreous cavity of the left eye that was initially interpreted as vitreous hemorrhage. Terson syndrome was the initial diagnosis but ophthalmoscopic examination and brain MRI showed that the left eye had silicone oil tamponade.* Conclusion*. Without knowing the history of previous vitreoretinal surgery, CT scan findings of intraocular silicone oil may be interpreted as vitreous hemorrhage. In patients with concomitant intracranial hemorrhage, it can masquerade as Terson syndrome.

## 1. Introduction

Vitreous hemorrhage following aneurysmal subarachnoid hemorrhage (SAH) was reported by Albert Terson in 1900 [1]. Terson syndrome appears to correlate with the extent of SAH and is associated with higher mortality rate [1].

Nowadays, Terson syndrome is described as any kind of intraocular hemorrhage in association with any type of intracranial hemorrhage [2]. Intraocular hemorrhage in Terson syndrome can take the form of vitreous, subhyaloid, or intraretinal hemorrhage. The probable mechanism is a sudden transient spike in intracerebral pressure that is transmitted along the optic nerve sheath, resulting in abrupt intraocular venous hypertension and subsequent rupture of retinal vessels [2]. Ocular complications including retinal detachment and proliferative vitreoretinopathy may occur following subhyaloid and vitreous hemorrhage. Early vitrectomy has been shown to be associated with better visual outcome and fewer complications [3].

Silicone oil is widely used for internal tamponade in the treatment of complicated retinal detachments. Silicone oil has unique characteristics in imaging modalities such as high attenuation on computed tomography scan (CT scan) and hyperintensity on T1-weighted magnetic resonance imaging (MRI) that can mimic intraocular hemorrhage [4]. Herein, we report a comatose patient with intracranial hemorrhage whose brain CT scan findings were suggestive of concomitant vitreous hemorrhage and hence patient was initially misdiagnosed of having Terson syndrome. Ophthalmic examination and brain MRI revealed the presence of silicone oil in vitreous cavity instead of hemorrhage.

## 2. Case Report

**Figure 1:**
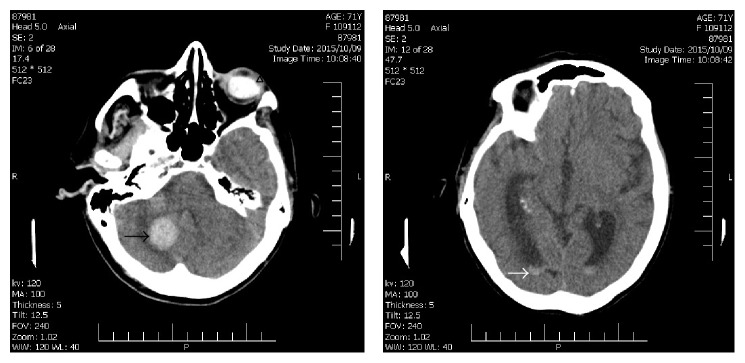
Brain CT scan. Hyperdensity in vitreous cavity of the left eye (arrowhead), hemorrhage in right cerebellum (black arrow), and intraventricular hemorrhage (white arrow).

A 71-year-old diabetic woman was admitted to the emergency center because of loss of consciousness. She had new onset headache, nausea, and vomiting a few hours before her admission. She had a history of controlled hypertension and previous myocardial infarction. On neurological examination GCS (Glasgow Coma Scale) was 9, and no neurologic localizing signs were detected. A brain CT scan was performed which revealed the presence of hyperdense lesions in right cerebellum and ventricles corresponding to cerebellar and intraventricular hemorrhage. A large hyperdense mass-like lesion was also noticed in left vitreous cavity which was assumed to be a vitreous hemorrhage (Figure 1).

Based on initial brain CT scan findings, a simultaneous intracranial and vitreous hemorrhage was assumed. With a diagnosis of Terson syndrome, an ophthalmology consultation was requested.

Handheld slit-lamp examination and indirect funduscopy revealed that the left eye was pseudophakic and had evidence of regressed diabetic retinopathy with extensive photocoagulation laser scars. There was no sign of vitreous or subhyaloid hemorrhage. However, the vitreous cavity of left eye was filled with silicone oil which indicated her previous vitreoretinal surgery. The right eye was pseudophakic with severe posterior capsule opacification making the fundus invisible.

**Figure 2:**
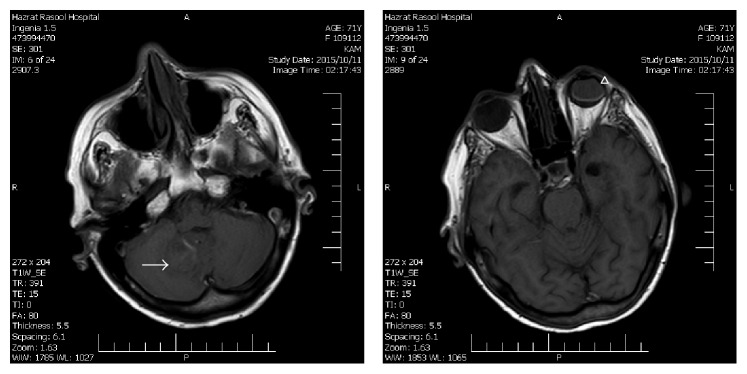
T1-weighted MR images. The intraocular silicone oil (arrowhead) is hyperintense relative to the vitreous of the fellow eye and isointense relative to the cerebellar hemorrhage (arrow).

**Figure 3:**
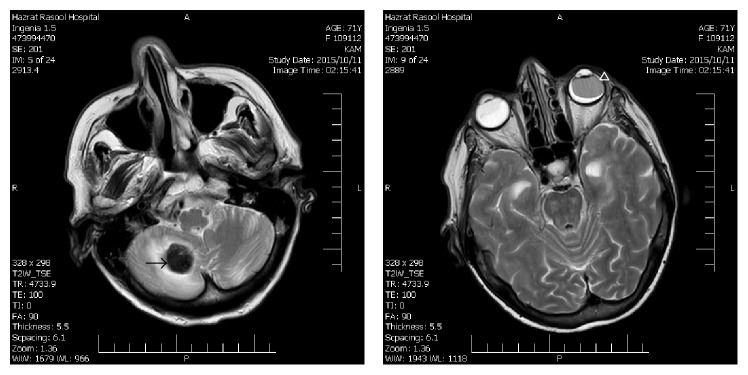
T2-weighted MR images. The intraocular silicone oil (arrowhead) is hypointense relative to the vitreous of the fellow eye and hyperintense relative to the cerebellar hemorrhage (arrow). Extensive retinal detachment is evident in the right eye.

48 hours later a brain MRI was performed as a routine diagnostic procedure. The follow-up MRI confirmed the presence of intracranial hemorrhage. Although T1-weighted images were also mimicking intraocular hemorrhage, T2-weighted images were in agreement with the presence of silicone oil rather than vitreous hemorrhage (Figures 2 and 3).

## 3. Discussion

Prompt and accurate diagnosis of Terson syndrome has clinical importance. Dilated fundoscopy is the method of choice for diagnosing Terson syndrome, but the debilitated state of many patients prevents them from noticing and expressing visual complaints. Besides, pharmacologic pupillary dilatation is not permitted in acute stage. This makes the detection of intraocular hemorrhage more challenging especially for nonophthalmologist physicians. Swallow et al. reported that clinical diagnosis of intraocular hemorrhage was often delayed for up to several months until discovery of a late visual defect [5].

Therefore, some investigators have tried to make the diagnosis of Terson syndrome based on evidences depicted by ancillary imaging modalities. CT scan has been shown to be a useful technique for detecting intraocular hemorrhage in patients with concomitant intracranial hemorrhage; it can show subhyaloid hyperdense crescents, hyperdense retinal thickening, and nodularity within the retina [5, 6]. Although a specificity of 97% has been reported for detection of Terson syndrome based on CT scan findings [6], false positive results are not uncommon. Previous studies have reported papilledema, globe position, bone artifacts, and beam hardening as the causes of false positive results [5–7]. The current report emphasizes the importance of considering intraocular silicone oil as another origin of false positive results in interpreting CT findings for detecting Terson syndrome.

## 4. Conclusion

Without knowing the history of previous vitreoretinal surgery, CT scan findings of intraocular silicone oil may be interpreted as vitreous hemorrhage. In patients with concomitant intracranial hemorrhage, it can masquerade as Terson syndrome.
